# Development and Validation of A Decision-Making Donor Conception Questionnaire in Iranian Infertile Couples

**DOI:** 10.22074/ijfs.2019.5700

**Published:** 2019-07-14

**Authors:** Fatemeh Hadizadeh-Talasaz, Masoumeh Simbar, Habibollah Esmaily, Robab Latifnejad Roudsari

**Affiliations:** 1 Department of Midwifery, Faculty of Medicine, Social Development and Health Promotion Research Center, Gonabad University of Medical Sciences, Gonabad, Iran; 2 Midwifery and Reproductive Health Research Center, Department of Midwifery and Reproductive Health, School of Nursing and Midwifery, Shahid Beheshti University of Medical Sciences, Tehran, Iran; 3 Social Determinent of Health Research Center, Mashhad University of Medical Sciences, Mashhad, Iran; 4 Nursing and Midwifery Care Research Center, Mashhad University of Medical Sciences, Mashhad, Iran; 5 Department of Midwifery, School of Nursing and Midwifery, Mashhad University of Medical Sciences, Mashhad, Iran

**Keywords:** Decision-Making, Donor Conception, Infertility, Validation

## Abstract

**Background:**

Despite the fact that many infertile couples have to decide about whether or not to choose donor con- ception, there is no predictive scale for evaluating the process of decision-making on donor conception and its deter- minants in such couples. The present study was conducted to develop a decision-making questionnaire for selecting donor conception and assess its psychometric properties in Iranian infertile couples.

**Materials and Methods:**

This cross-sectional validation study was conducted based on the method developed by DeVellis (2012) in four steps at Milad Infertility Clinic, Mashhad, Iran. The dimensions of the concept of decision- making were determined in the first step based on the qualitative results obtained from 38 semi-structured in-depth interviews. Items that were appropriate for the questionnaire were developed in the second step using the qualitative data and a review of the literature. In the third step, the research team reviewed and eliminated some of the items. The fourth step evaluated the face, content and construct validity of the questionnaire through exploratory factor analysis on a sample of 220 infertile couples using convenience sampling and investigated its initial and final reliability.

**Results:**

Based on the results of the qualitative study, a pool of 170 items was developed, 101 of which were elimi- nated after revision due to ambiguity, repetition or their poor face and content validity and initial reliability. The questionnaire was evaluated for its construct validity with 69 items. After the exploratory factor analysis, the decision- making donor conception questionnaire (DMDCQ) having 51 items and seven factors, was finalized. All the factors had Cronbach’s alpha values of 0.75-0.87 and intra-class correlation coefficients (ICC) greater than 0.7.

**Conclusion:**

This study led to development of a valid and reliable scale for examining infertile couples’ decision- making about whether or not to use donor conception as well as the determinants of this decision.

## Introduction

Advances in assisted reproductive technology (ART) offer new methods of getting pregnant and make parenthood possible for people deprived of having children for various reasons (1). Although these technologies are a ‘marriage saver’ for those left without a child (2), give hope to millions of infertile couples (3, 4) and help them to realize their dream of raising a family (5), not all infertile couples use reproductive technologies (6) and the demand for these treatments is unexpectedly low (7). In fact, only half of infertile couples around the world seek treatments (7, 8). Deciding whether or not to use these technologies is definitely difficult (9), and many sociocultural, ethical, legal and religious challenges surrounding different aspects of ART, such as donor conception, can affect the practical use of these technologies (3, 4).

Deciding to use these technologies is influenced by people’s perceptions and the society’s expectations and attitudes toward their use (6). In other words, sociocultural beliefs affect couples’ tendency toward using these methods (10, 11) and influence the rate of employment of these technologies by couples (12). Infertile couples who have a child born through donor conception, experience great prejudice not only by the society but also by their family, relatives and friends. In developing countries, the family’s rejection and social pressures are among the factors affecting the decision about seeking a method of treatment and the choice of treatment is made under the heavy influence of family members (13). Many infertile couples suffer from the stigma of infertility and seeking treatment, and try to keep their condition a secret (14). They feel that they will be ethically judged for their infertility and their decision to use ART (15). The individual’s beliefs and attitudes may be the most important determinant of his/ her actions. Individuals with strong spiritual beliefs and specific sociocultural beliefs may adopt approaches and treatment methods that are different from those adopted by other infertile individuals, and their use of donor conception is also influenced by different factors, as they attribute different meanings to their condition and its treatment and interpret them differently (16). Some infertile couples for whom donor conception is the only way of becoming parents, they might prepare themselves for a childless life or accept to adopt a child and reject medical treatments. Some others, in contrast, try all the available treatments in different medical centers and greatly invest for this goal both in material and emotional terms (17). Sociocultural beliefs may also affect people’s religious beliefs (18). In other words, cultural factors can reinforce or inhibit religious attitudes toward the use of ART. Religion also plays a major role in the use of ART, as it affects people’s views and social norms. It is difficult to have access to ART in countries with religious dogmatism (2). The decision on the employment of ART is made according to the laws of the society (19). Laws have a significant effect on the access to ART (2). In some countries, donation is a process, while in others, there are limited rules. In New Zealand, embryo donation is a key process that is based on rules and policies (20), while in Australia, there are few rules about the donation process (21). Laws are largely based on the sociocultural state of the society and its ethical, spiritual and religious values (19, 22). The limited number of donors is also one of the main practical factors affecting most couples’ decision about the selection of a donor (23). Economic issues also affect the access to ART (24).

Deciding about the use of donor conception services is therefore a complicated and difficult process for couples which challenges their values and beliefs. Making this decision is a complicated social and interactive process that is under the influence of various individual, social, economic, cultural, psychological and ethical factors and is affected by the couple’s interactions with each other and with their family, friends, health workers, key people, etc. It is therefore necessary to develop a scale for identifying the determinants of infertile couples’ decision about using donor conception to perform supportive interventions that improve the decision-making process and reduce the outcomes of the decision including regret. A review of the literature did not show any instruments developed for direct measurement of the subject in question. Given the complexity of the decision-making process about this issue and the absence of an instrument for its assessment, the present study was conducted to fill the gap, develop a decision-making for donor conception questionnaire (DMDCQ) and determine its psychometric properties in Iranian infertile couples.

The scale developed in this study measures the determinants of infertile couples’ decision-making and can help specialists to understand the issues around infertile couples’ decision making concerning the use of ART and design individual and public training programs and instructional decision-making packages for resolving the barriers and thus reducing the need for unnecessary interventions.

## Materials and Methods

This cross-sectional validation study was performed using the method developed by DeVellis in 2012 (25) in four steps, after combining some of the stages: 

### First step: Performing a qualitative study and extracting the dimensions or constructs of the intended concept

In the first step, the concept under measurement (i.e. decision-making for donor conception) was theoretically defined. For the first step and in order to explain participants’ experiences regarding the process of decision- making for donor conception, a qualitative study with a grounded theory approach was performed in 2014 in Mashhad, Iran, using individual interviews. A total of 38 participants including nine eligible infertile couples (four couples who were candidates for receiving egg donation, three couples candidates for receiving embryo donation, one couple candidate for receiving egg and uterus donation and one couple candidate for receiving uterus donation) and 14 eligible women (seven egg donor candidates, four embryo donor candidates, one egg and uterus donor candidate and two uterus donor candidates), were enrolled. The key people involved in decision-making for donor conception, including two gynecologists, two midwives and two clergymen, were also interviewed during the theoretical sampling, and this process was continued until the saturation of the categories without any restrictions on the number of participants and according to the theoretical requirements of the study. 

The inclusion criteria were being married, Iranian, and infertile (either male or female infertility or both), having no biological or adopted children, nor other spouses, having the experience of using at least one ART in the past or being under treatment with ART or in the waiting list to receive ART, being willing to participate in the study and being able to communicate and express their experiences. The selected members of the infertility treatment team had at least one year of experience of working with infertile couples. The selected clergymen were experts in this field and were interested in participating in the study. The study was performed at Milad Infertility Clinic, Mashhad, Iran. The participants were selected through purposive convenience sampling with maximum variation in terms of age, duration of infertility, duration of treatment, education and socioeconomic status. Sampling was continued until the saturation of the data. Data collection was mainly done through semi-structured in-depth interviews directed by the interview guide, that enabled the participants to freely discuss the matter. All interviews were done by one of the researchers. The interviews were conducted separately with the infertile men and women, but a couple interview was also held with both the husband and wife if there was an obvious difference in their answers. Each interview took 40-120 minutes and was held in one or more sessions. The interviews were recorded with participants’ permission. Data were analyzed concurrently using MAXQDA-2007 and five dimensions ultimately emerged. The approval of the local Research Ethics Committee of Shahid Beheshti University of Medical Sciences was obtained along with the informed consent of all participants before beginning the study.

### Second step: Producing an item pool using an inductive method

In the second step, an item pool was produced using an inductive method; for this purpose, items relevant to the main concepts of donor conception decision-making were developed based on the qualitative findings of the study (n=170). Participants’ attitude toward each item was measured on a 5-point Likert scale from “quite agree” to “quite disagree”.

### Third step: Initial items reduction

In the third step, the initial items extracted from the qualitative study were reviewed by the research team and the repetitive and ambiguous items were removed. Eventually, 113 items were developed in five dimensions, including being offered to use donor conception (10 items), inner turmoil (4 items), attempts for coping with the current conditions (23 items), deciding to accept and use donor conception (54 items) and deciding to undergo treatment (22 items).

### Fourth step: Validation of the questionnaire through assessing its face validity, content validity, initial reliability, construct validity and final reliability

The face validity of the questionnaire was evaluated both qualitatively and quantitatively in the fourth step. To perform the qualitative evaluation, face-to-face interviews were conducted with ten similar members of the target group (four infertile men and six infertile women who met the inclusion criteria) and difficulties in understanding the words and phrases, the degree of inappropriateness of the phrases or their irrelevance to the questionnaire dimensions, ambiguities causing misunderstanding of the phrases, or the words failing to convey a meaning, were examined. Once the items were modified according to the received feedback, the item impact was measured quantitatively. The objective in this step was to determine the item impact score in a sample that was similar to the target group. For this purpose, each item was scored on a 5-point Likert scale as follows: 5: “quite important”, 4: “somewhat important”, 3: “relatively important”, 2: “slightly important”, and 1: “not important at all”. Ten individuals similar to the target group (four infertile men and six infertile women who met the inclusion criteria) were asked to determine the importance of each item based on their own experiences. The researcher calculated the impact score (IS) for each item separately based on the following equation (26):

Impact score=Frequency percentage×level of significance

Frequency percentage=The percentage of all the people who have reviewed each item

The items with an IS <1.5 were considered inappropriate and removed from the questionnaire (26).

The content validity of the questionnaire was evaluated both qualitatively and quantitatively. For the qualitative assessment of the content validity, the questionnaire was distributed among ten specialists (Ph.Ds in reproductive health or health education, and a number of gynecologists) and they were asked to give their feedback on the questionnaire. The content validity ratio (CVR) and content validity index (CVI) were used for the quantitative assessment of the content validity.

To determine the CVR, ten specialists were asked to review each item on a 3-point scale (3: necessary, 2: useful but not necessary, and 1: not necessary). The CVR was then calculated based on Lawshe’s formula as follows (27-29).

CVR=(ne–N/2)/(N/2)

ne: The number of specialists who have selected the “necessary” response

Based on Lawshe’s Table of minimum values, items with a CVR >0.62 as per the evaluation of the ten specialists, were deemed significant (P<0.05) and remained in the questionnaire (27-29).

The CVI for each item was examined based on the Waltz and Bausell CVI and the three criteria of simplicity, specificity (relevance) and clarity were separately measured on a 4-point Likert scale by the ten specialists. To calculate the CVI for each item, the total number of specialists who had given 3 and 4 points (i.e. the highest score) to that item was divided by the total number of specialists (n=10). The items with a CVI >0.79 were deemed acceptable (27-29). The items with a CVI of 0.7-0.79 were reviewed by the researcher and discussed again with the specialists. The items with a CVI <0.7 were eliminated from the questionnaire (30).

After determining the face and content validity, the initial reliability was calculated as the item analysis index. For this purpose, 30 infertile men and women visiting the infertility clinic were selected by convenience sampling to complete the initial questionnaire, and the Cronbach’s alpha was calculated to determine the internal consistency for each factor as well as the entire scale. Cronbach’s alpha values of 0.7 were considered favorable in this study.

The construct validity was determined by exploratory factor analysis. For analysis of the data, the exploratory factor analysis was performed in seven steps: determining the sample size, examining the correlation between the items, deciding about the items being fit for the factor analysis, determining the number of initial factors extracted, rotating and extracting the final factors and naming the factors.

According to Tabachnick and Fidell (31), evaluation of the construct validity requires a sample size that is three to five times larger than the number of items in the scale. Given the number of items in the final questionnaire (i.e. 69) and the potential sample loss, 220 subjects were included in this study. The inclusion criteria consisted of being married, Iranian, infertile (with male and/ or female infertility) and candidate for ART [intrauterine insemination (IUI), in vitro fertilization (IVF), gamete intrafallopian transfer (GIFT), and intracytoplasmic sperm injection (ICSI)], and having enough information about donor conception.

The correlation between each item and the other items was examined by principal component analysis (PCA), and the items that had correlation with the other items of <0.3, were eliminated from the analysis.

**Fig 1:**
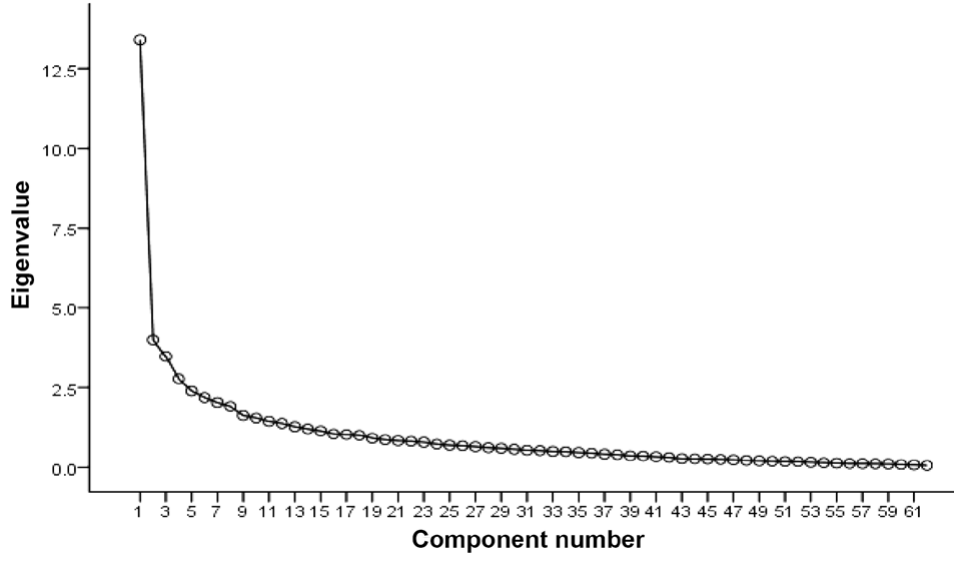
Scree plot.

The Kaiser-Meyer-Olkin (KMO) measure of sampling adequacy was used to ensure the adequacy of the samples. If the KMO measure is >0.70, the set of data is deemed fit for factor analysis. Bartlett’s test of sphericity was also used to examine the fit of the data for the factor analysis. If the P value is <0.05 in this test, factor analysis is considered an appropriate technique (32). The community statistic was used to detect inappropriate items whose variance was not used for explaining the variance of the main factor. In this study, the inflection point of 0.4 was taken as the minimum factor loading required for keeping each item in the factors extracted through the factor analysis. To extract the required number of factors, a scree plot (Fig .1) and eigenvalues were used and the percentage of variance of each factor was calculated. The factors with eigenvalues >2 remained in the study. The final factors were extracted by varimax rotation. 

The reliability of the questionnaire was examined using the internal consistency and test-retest stability methods. To measure the internal consistency, 30 infertile men and women visiting Milad Infertility Clinic were selected by convenience sampling to complete the questionnaire, and Cronbach’s alpha values were calculated for each factor and the entire questionnaire. Cronbach’s alpha values of =0.7 were deemed acceptable. To determine the stability of the questionnaire, 20 infertile men and women completed the questionnaire within a two-week interval and the intraclass correlation coefficient (ICC) was then calculated. An ICC >0.70 was deemed acceptable (33).

## Results

A total of 220 infertile men and women who met the inclusion criteria participated in this psychometric assessment. Table 1 presents the demographic and infertility characteristics of the participants. 

**Table 1:** The demographic and infertility-related characteristics of the participants


Participants’ characteristics		n (%) or
n=220		mean ± SD

Sex		
	Male	55 (25)
	Female	165 (75)
Education		
	Below high school diploma	50 (22.7)
	High school diploma	72 (32.8)
	Associate or bachelor’s degree	86 (39.1)
	Master’s degree or higher	6 (2.7)
	No answers	6 (2.7)
Age (Y)		29.7 ± 5.08
Infertility duration (month)		63.4 ± 43.2
Treatment duration (month)		34 ± 32.5
Infertility cause		
	Ovarian	64 (29.1)
	Uterine	6 (2.7)
	Ovarian and uterine	15 (6.8)
	Tubal	12 (5.5)
	Endometriosis	6 (2.7)
	Male factor	56 (25.5)
	Unknown	41 (18.6)
	No answers	20 (9.1)
Family history of infertility		
	Yes	79 (35.9)
	No	137 (62.3)
	No answers	4 (1.8)


SD; Standard deviation.

Based on the results of the qualitative content analysis, an item pool was composed of 170 items, and the ambiguous and repetitive items were removed after the revision done by the research team. Eventually, 113 items were developed in five dimensions or constructs, including being offered to use donor conception (ten items), inner turmoil (four items), attempts for coping with the current conditions (23 items), deciding to accept and use donor conception (54 items) and deciding to undergo treatment (22 items), which entered the psychometric assessment phase. The evaluation of face validity, which was performed qualitatively and quantitatively, led to the removal of eight items, and the questionnaire entered the content validity evaluation stage with 105 items. The content validity was also evaluated both qualitatively and quantitatively and 28 items were removed, leading to the existence of 77 items. In the stage of initial reliability evaluation, the Cronbach’s alpha was calculated separately for each item, eight items were removed, and the remaining 69 items entered the construct validity evaluation stage. It should be noted that the questionnaire’s reliability increased to over 0.7 once these items were removed, and according to the researcher, their removal did not destroy the basic information required. The initial Cronbach’s alpha calculated for the entire scale was 0.82. 

To determine the construct validity of the scale, 220 participants were selected through convenience sampling to complete the questionnaire. There was no sample dropout. The collected data were entered into SPSS-22. The PCA showed that the correlation between two of the items and the other items was <0.3; thus, both of these items were removed and the factor analysis was continued with 67 items. The KMO measure for the items was 0.768, which indicates the sampling adequacy. The Bartlett's test of sphericity showed the fit of the data for the factor analysis with P<0.001. The community statistic was >0.4 for most of the items in this study and the items were thus considered fit for factor analysis. Five items with a community statistic <0.4 were excluded from the study, and the factor analysis was continued with 62 items. Determining the number of factors constructing the questionnaire using the factor analysis of the items led to the identification of seven factors with eigenvalues >2 and explaining 48.796% of the total variance. The items were rotated and categorized in each factor using a varimax rotation. Of the 62 items that entered the factor analysis in this study, 51 items and seven factors remained.

The factors were named based on the meaning of their items, especially the meaning of the item with the maximum factor loading, and with regard to the correlation found between the items and the available theoretical knowledge. The researcher referred to the qualitative part of the study and the categories and subcategories forming each item, in order to name the factors (Table 2).

**Table 2:** The factor loading of the Desision-Making for Donor Conception Questionnaire items in Iranian infertile couples


Factor																							
	1			2			3			4			5			6			7		

**Factor 1: The role of social networksIt is difficult for me to accept donor conception because of people’s negative attitude toward this method.**			0.638								
The treatment team’s commitment to keep my information confidential is important to me to accept donor conception.			0.622								
The positive experiences of people who have used donor conception affect my decision to accept this method.			0.608								
The treatment team’s honesty in explaining the cause of infertility affects my decision to accept this method.			0.577								
The infertility clinics’ provision of clear information about the costs of donor conception affects my decision to accept this method.			0.559								
I need more time for making a decision to accept this method.			0.558								
I may have to accept donor conception in order to save my marriage.			0.554								
Clergymen’s approval of donor conception helps me to decide about these methods more quickly.			0.552								
The existence of laws about donor conception affects my decision to accept this method.			0.542								
The society’s familiarity with donor conception helps me to accept this method easier.			0.531								
Consulting sessions held before and during treatment with donor conception affect my decision-making.			0.469								
The failure to provide clear and proper information about donor conception affects my decision to accept this method.			0.459								
I may have to accept donor conception in order to free myself of other people’s babble.			0.455								
Other people’s refraining from interfering in our childbearing or way of childbearing affects my decision to accept this method.			0.427								
**Factor 2: Coping strategiesWhen offered to use donor conception, practices such as praying can make me calm and enable me to make a more rational decision**				0.782							
The belief in God’s will makes me peaceful and affects my decision about whether or not to accept this method				0.718							
When offered to use donor conception, changes in lifestyle, such as working more, make me think less about my problem and make a more rational decision.				0.539							
When offered to use donor conception, thinking about positive issues makes me calmer and enables me to make a more rational decision.				0.535							
**Factor 3: The decision to disclose or concealIf I use donor conception, I won’t inform others of my decision because I fear that my child may accidentally learn of the matter from them.**					0.760						
The possibility of concealing the matter from others affects my decision about whether or not to accept donor conception.					0.714						
If I use donor conception, I won’t inform others of my decision, because I fear their negative reaction (blaming, humiliation and ridicule) toward myself and my child					0.688						
If I decide to use donor conception, I will hide it from my child.					0.681						
If I decide to use donor conception, I may change my job or address					0.558						
If I decide to use surrogacy services, I will try to pretend to be pregnant.					0.526						
If I decide to use donor conception, I will inform my first-degree relatives (mother and sister) in order to get support from them.					0.422						
**Factor 4: Interpersonal relationships**									
I feel that if I decide to use donor conception, my emotional relationship with my husband might suffer						0.738					
I feel that if I decide to use donor conception, my sex life might suffer.						0.726					
If I decide to use donor conception, I reduce my relationships with others						0.575					
**Factor 5: Religious quests**									
If I decide to use donor conception, I won’t inquire into the religious aspects of using these methods, because they are being performed in official infertility clinics in an Islamic country							0.564				
If I decide to use donor conception, I will ask people who have previously used these methods about its religious issues							0.464				
If I decide to use donor conception, I will seek the fatwa of other religious references in order to reach my goal of having a child, if my own religious reference opposes this method.							0.417				
**Factor 6: Donor’s characteristics**									
If I decide to use donor conception, the donor’s characteristics won’t matter much to me; the only thing that will matter to me is to find the donor faster								0.802			
If I decide to use donor conception, I won’t inquire much into the donor’s background, because I have to accept her with any conditions due to the limited number of donors								0.731			
If I decide to use donor conception, I will prefer to use the services of a donation center in order to avoid future disturbances by the donor								0.696			
If I decide to use donor conception, I will try not to inquire much into the donor’s background, because it may dishearten her and make her change her mind								0.695			
If the decision to use donor conception becomes certain, I will prefer a known donor because of her availability and the shorter waiting time								0.580			
If I decide to use donor conception, I will prefer to use donation centers because I can access the donor faster that way								0.576			
If the decision to use donor conception becomes certain, I will inquire greatly into the donor’s background before selecting her								0.483			
If I decide to use donor conception, the donor’s moral health will be the most important selection criterion for me								0.477			
If I decide to use donor conception, I will prefer an unknown donor because I fear others’ learning of my decision								0.429			
**Factor 7: Challenges in the process of treatment**									
If I decide to use donor conception, the unavailability of a donor will be one of the main barriers									0.642		
A better coordination between infertility clinics and the legal authorities shortens the duration of the legal procedures and accelerates the decision to use donor conception									0.567		
If I decide to use donor conception, I will use a method that best fits my mental conditions									0.604		
The lengthy and time-consuming stages of donor conception make me delay the decision to undergo this treatment									0.599		
If I decide to use donor conception, I will choose a clinic that costs less									0.572		
The support of others (including my spouse and family) accelerates my decision to use donor conception									0.565		
If I decide to use donor conception, I will choose a clinic that has served longer and has more experienced personnel									0.551		
The high cost of treatment is a barrier to my decision to use donor conception									0.638		
If I decide to use donor conception, I will use a method that has the shortest waiting time									0.488		
The availability of medical facilities at nearby infertility clinics accelerates my decision to use donor conception									0.481		
If I decide to use donor conception, I will try to resolve the barriers with various solutions									0.449		


Table 3 summarizes the number of items in each subscale and the range of scores for the entire DMDCQ and its subscales. 

**Table 3:** The range of scores for the total and subscales of the DMDCQ


Subscale	Number of items	Range of scores

Role of social networks	14	14-70
Coping strategies	4	4-20
The decision to disclose or conceal	7	7-35
Interpersonal relationships	3	3-15
Religious quests	3	3-15
Donor’s characteristics	9	9-45
Challenges in the process of treatment	11	11-55
Total	51	51-255


Table 4 summarizes the mean and standard deviation of the total and subscale scores of the DMDCQ in the entire sample of participants. When the total score of the questionnaire and the scores of its subscales are higher, higher numbers of individuals make positive decisions and the couple will be more inclined toward donor conception in the future. 

**Table 4:** The mean and standard deviation (SD) of the total and subscale scores of the decision-making donor conception questionnaire (DMDCQ) in the entire sample (n=220)


Subscale	Mean ± SD	Min	Max

Role of social networks	53.57 ± 8.63	22	66
Coping strategies	17.75 ± 2.48	4	20
The decision to disclose or conceal	24.75 ± 5.16	11	35
Interpersonal relationships	8.41 ± 1.97	3	15
Religious quests	10.14 ± 2.76	3	15
Donor’s characteristics	30.21 ± 5.26	9	45
Challenges in the process of treatment	43.20 ± 7.78	15	55
Total	188.50 ± 22.27	115	235


Min; Minimum and Max; Maximum.

The initial Cronbach’s alpha was 0.82 for the entire scale and 0.75-0.87 for each subscale. The ICC was >0.7 for all the factors, which confirms the high reliability of the questionnaire (Table 5).

**Table 5:** The Cronbach’s alpha and intraclass correlation coefficient (ICC) of subscales and the entire questionnaire



Subscales	Cronbach’s alpha	ICC
Role of social networks	0.85	0.96
Coping strategies	0.79	0.80
The decision to disclose or conceal	0.83	0.91
Interpersonal relationships	0.75	0.78
Religious quests	0.76	0.84
Donor’s characteristics	0.79	0.95
Challenges in the process of treatment	0.87	0.88
Total	0.82	0.86


## Discussion

The questionnaire developed in this study is the first and only valid and reliable scale developed and psychometrically assessed in the world, concerning donor conception decision-making. The questionnaire consists of 51 items within seven factors, including the role of social networks, coping strategies, the decision to disclose or conceal, interpersonal relationships, religious quests, donor’s characteristics and challenges in the process of treatment. These seven factors explained 48.796% of the total variance.

A review of the literature showed that no specific scale was developed for donor conception decision-making for infertile couples. Decision-making scales such as Flinders’ decision-making questionnaire and the Melbourne decision-making questionnaire with different numbers of constructs, mostly address general issues.

Flinders’ decision-making questionnaire was developed in 1982 by Mann, for the measurement of coping patterns and strategies for decision-making in conflict resolution and consists of 31 items and three constructs, namely vigilance, hyper vigilance and defensive avoidance (including procrastination, buck-passing and rationalization). Mann et al. (34) examined the construct validity (confirmatory) of Flinders’ decision-making questionnaire in different cultural contexts (i.e. in the United States, Australia, Japan, Hong Kong, Taiwan and New Zealand). They eliminated the rationalization factor because it was not a good fit for the model and developed a new questionnaire called the Melbourne decision-making questionnaire, consisting of 22 items and four constructs, including vigilance, hyper vigilance and procrastination and buck-passing, and it replaced Flinders’ decision-making questionnaire. Although the “rationalization” construct was eliminated from Flinders’ decision-making questionnaire through the confirmatory factor analysis, coping strategies (including the use of rationalization and relaxation strategies) comprise an important factor of the DMDCQ, perhaps owing to the special nature of donor conception decision-making for infertile couples or because of the differences in the cultural contexts examined. A number of items from the Melbourne decision-making questionnaire was incorporated into the various items of the DMDCQ, such as the item “I may have to accept donor conception in order to free myself of other people’s babble”, which is similar to the item “I do not decide unless I really have to” in the Melbourne decision-making questionnaire.

Decision-making instruments about health issues include the decisional conflict scale (DCS), which measures decisional conflict in patients and contains 16 items and three subscales, including uncertainty in making a health-related decision, modifiable factors contributing to uncertainty and perceived effective decision making (35). This scale was translated into Dutch, French and Spanish and psychometrically assessed (36). Some of the items in the DCS have been incorporated into the various items of the DMDCQ, such as the item “The support of others (including my spouse and family) accelerates my decision to use donor conception”, which is similar to the item “I have enough support from others to make a choice” in the DCS. A difference between the two scales is that one of the subscales in the DCS is about perceived effective decision-making, which indicates the user’s degree of agreement about the informed decision, its compatibility with her personal values and her satisfaction with her decision. The scale developed in the present study, however, lacks a similar factor. 

The decision-making scale for women with unplanned pregnancy is another decision-making scale in gynecology, which was developed by Nourizadeh et al. (37). This questionnaire consists of two scales that measure two important concepts of decision-making in women with unplanned pregnancy. The first scale measures the concept of perceived threats and is composed of 33 items within six factors, including fear of anomalies and violation of the norms, fear of difficulty and the aggravation of instability, fear of parental responsibility and commitments, fear of abortion and escape from abortion, role conflicts and social deprivations, and fear of negative physical-emotional consequences. The second scale measures decision-making style and strategies in women with unplanned pregnancy and consists of 27 items within four factors, including resistance against acceptance, avoidance-justification strategies, analytical strategies and confirmatory strategies (37). Coping strategies (the use of rationalization and relaxation) comprise an important factor of the DMDCQ that is similar to the decision-making scale for women with unplanned pregnancy, in which justification strategies (rationalizing to oneself and others) also comprise an important factor. Some of the items in the decision-making scale for women with unplanned pregnancy have been incorporated into the various items of the DMDCQ, such as the item “If I use donor conception, I won’t inform others of my decision, because I fear their negative reaction (blaming, humiliation and ridicule) toward myself and my child”, which is similar to the item “I have hidden my pregnancy from others because I am inclined toward abortion and fear others’ objection or obstruction of abortion” in the decision-making scale for women with unplanned pregnancy. The review of items showed that both scales emphasize the role of social norms in decision-making in a way that the violation of norms is a barrier to decision-making. Consequently, people who decide to use donor conception may try to conceal it in order to avoid others’ blames. A difference observed between these two scales is that confirmatory strategies comprised one of the factors in the decision-making scale for women with unplanned pregnancy, which is concerned with others’ approval and indicates counseling for the purpose of making a rational and acceptable decision. The instrument developed in the present study, however, does not include such constructs.

The general strengths of the questionnaire developed in this study include its specificity and its ease of completion. The average time taken to complete the questionnaire was 10-15 minutes depending on the respondent’s literacy. 

One of the limitations of this study was the limited number of samples applying for donor conception in the only governmental infertility center in Mashhad. Other limitations included sampling from the men, as some of their wives opposed to be interviewed. Also, due to the uniqueness of the study tool, it was not possible to compare the results with other countries or check the tool’s empirical validity. Respondent bias was another limitation of this study.

## Conclusion

The DMDCQ can contribute to the development of an instructional decision-making package and supportive interventions for improving processes of decision-making and reducing negative physical and psychological outcomes and regrets by informing caregivers and counsellors about the circumstances and procedures of decision- making by couples. 
